# Role of Lateral Inhibition on Visual Number Sense

**DOI:** 10.3389/fncom.2022.810448

**Published:** 2022-06-20

**Authors:** Yiwei Zhou, Huanwen Chen, Yijun Wang

**Affiliations:** The School of Automation, Central South University, Changsha, China

**Keywords:** lateral inhibition, number sense, size effect, distance effect, neural network model

## Abstract

Newborn animals, such as 4-month-old infants, 4-day-old chicks, and 1-day-old guppies, exhibit sensitivity to an approximate number of items in the visual array. These findings are often interpreted as evidence for an innate “number sense.” However, number sense is typically investigated using explicit behavioral tasks, which require a form of calibration (e.g., habituation or reward-based training) in experimental studies. Therefore, the generation of number sense may be the result of calibration. We built a number-sense neural network model on the basis of lateral inhibition to explore whether animals demonstrate an innate “number sense” and determine important factors affecting this competence. The proposed model can reproduce size and distance effects of output responses of number-selective neurons when network connection weights are set randomly without an adjustment. Results showed that number sense can be produced under the influence of lateral inhibition, which is one of the fundamental mechanisms of the nervous system, and independent of learning.

## Introduction

Lateral inhibition is widespread in the animal nervous system, one of the fundamental mechanisms of information processing in the brain, and is present in the primary visual cortex (Lu and Zuo, 2017) and neocortex (Zhou and Yu, 2018) associated with visual processing. Excitatory neurons inhibit the activity of surrounding neurons when neurons in these cortical areas are stimulated. The intensity of inhibition decays with the increasing distance between neurons and results in a “Mexican hat” interaction relationship (Field et al., 2020). This interaction can disrupt the attractor state and produce rich responses to encode different characteristics of external stimuli, such as duration, intensity, and number (Miller, 2013). A previous study showed that the width of lateral inhibition can affect the encoding mode of number-selective units (Chen et al., 2020). Many studies have also shown that lateral inhibition contributes to the generation of number sense in animals (Miller, 2013; Cappelletti et al., 2014; Sengupta et al., 2014). Therefore, adding lateral inhibition to the model and discussing the influence of lateral inhibition on the number sense of the model are necessary.

Many number sense models based on artificial neural networks directly or indirectly use lateral inhibition function and can qualitatively or quantitatively reproduce the results of biological experiments (Dehaene and Changeux, 1993; Sengupta et al., 2014; Nieder, 2016; Hannagan et al., 2017; Nasr et al., 2019). However, these models fail to explain the role of lateral inhibition systematically in the production of number sense due to the following reasons. First, the majority of models only use lateral inhibition without analyzing its role (Dehaene and Changeux, 1993; Hannagan et al., 2017; Nasr et al., 2019; Chen et al., 2020). Second, human beings can estimate the number of items (i.e., numerosity) without visual training. However, some models must be trained to realize the number sense; therefore, these models are unsuitable for analyzing the impact of lateral inhibition on number sense (Nieder, 2016; Burr et al., 2017). Third, some researchers use deep convolutional neural network calculation models with complex structures to solve the numerical cognition problem in complex real visual scenes (Nasr et al., 2019). Analyzing the effect of lateral inhibition alone is difficult due to the large number of hyperparameters and parameters in these models. Therefore, establishing a model with a simple network structure and lateral inhibition function is necessary to investigate the role of lateral inhibition in the generation of number sense from the perspective of function.

We built a number sense model on the basis of lateral inhibition to investigate whether neonatal animals present an innate “number sense” and determine important factors that affect the number sense. The dataset was inputted into the untrained model (i.e., randomly set network connection weights without adjustment), and the output response was observed to assess whether the model presents the number sense. Based on this, the role of lateral inhibition in each layer of the neural network was analyzed by canceling the lateral inhibition function.

## Materials and Methods

### Stimulus Datasets

**FIGURE 1:**
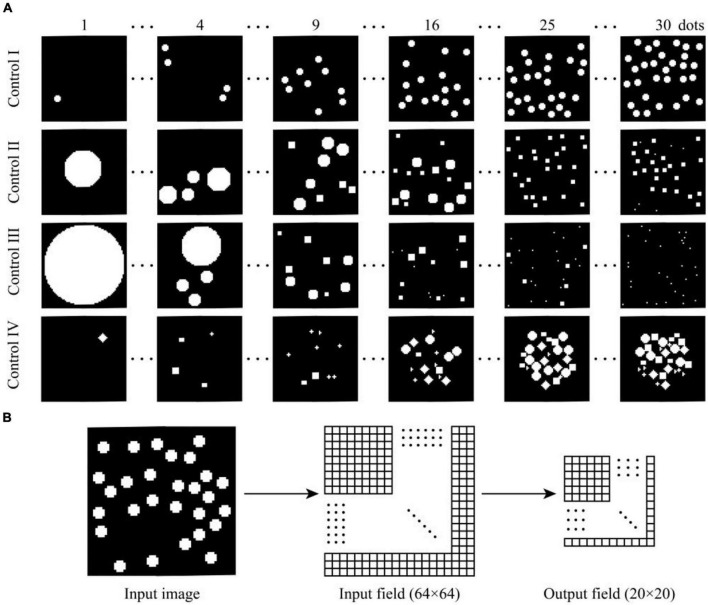
Schematic of the stimulus set and neural network structure. **(A)** Examples of four stimulus sets used to assess the number sense. **(B)** Number sense model based on lateral inhibition.

We constructed four different stimulus sets to test the response of the model to different numerosities and exclude the influence of non-numerical visual stimulus cues. The area and perimeter of each item were maintained at 21 and 12 pixels, respectively, in the first control set. The total area of items was maintained at 600 pixels in the second control set. The total perimeter of items was maintained at 180 pixels in the third control set. Convex hull of items was a regular pentagon with an outer circle diameter of 60 pixels and shapes of individual items varied in the fourth control set, with possible shapes of cross, rectangle, circle, triangle, square, and diamond. Four control sets are established to test whether the response of the model will change when items are distributed centrally rather than uniformly on the image and examine the influence of the item shape on the model response. Each image in the stimulus set contained 64×64 pixels, and the stimulus intensity of each pixel ranged from 0 to 1. Each control set consisted of 30 images with *n* = 1, 2, 3, 4, 5, 6, …, 30 items. Thirty images of the control set were inputted into the model, and the response curve of each unit was recorded when testing numerical capabilities of the model. This process was repeated 30 times, and weights of the model and 30 images included in the control set were randomly regenerated each time. The average tuning curve of each numerosity-selective unit was calculated by averaging the response of each unit to the same numerosity in 30 repeated runs. Four different stimulus sets are shown in Figure 1A.

### Lateral Inhibition Model

The programming language Python was used on the open-source machine learning platform TensorFlow to build a two-layered number sense model on the basis of lateral inhibition (Figure 1B). The network size of the input layer is 64×64 pixels, and each unit corresponds to one pixel of the input image. The network size of the output layer is 20×20*pixels*. Units located in different layers are fully connected, and initial weights follow the Gaussian distribution of μ = 0.5 and σ^2^ = 0.1. This distribution pattern is qualitatively similar to the synaptic weight distribution observed in biological experiments (Peng et al., 2017).

All units simultaneously inhibited one another in the same layer after the input layer received the image stimulus or the output layer received the input layer stimulus. The inhibition intensity is a function of the Euclidean distance *R* between units and the unit output intensity *a*_*x,y*_ that conforms to characteristics of the Gaussian curve. Hence, the inhibition intensity between units decreases with the increase of *R*.

The lateral inhibition process of input and output layers is expressed as follows:


(1) $$a_{x_{0},y_{0}}^{i\,n\,h}=a_{x_{0},y_{0}}-\sum_{x=1}^{\text{rows}}\sum_{y=1}^{\text{cols}}e^{-\frac{R^{2}}{2\times \sigma_{x,y}^{2}}}\times a_{x,y}^{2}\left(x\neq x_{0},y\neq y_{0}\right),$$

where *a*_*x_0,y_0*_ is the output of the unit located in row *x_0* and column *y_0* in the input or output layer before lateral inhibition, $a_{x_{0},y_{0}}^{i\,n\,h}$ represents the output of the unit after lateral inhibition, and *a*_*x,y*_ is the output of the unit located in row *x* and column *y* in the input or output layer before lateral inhibition. The intensity of lateral inhibition should be higher than that of the excitatory stimulus, given that the neural network must avoid overexcitation (Scharfman and Brooks-Kayal, 2014; Hattori et al., 2017). Therefore, the inhibitory intensity of any unit on surrounding units was set to be two times as strong as its input stimulus *a*_*x,y*_. *R* represents the Euclidean distance between units *a*_*x_0,y_0*_ and *a*_*x,y*_, and σ_*x*,*y*_ is the standard deviation that determines the range of lateral inhibition of the unit located in row *x* and column *y* in the input or output layer. The lateral inhibition range of each neuron in the cerebral cortex is different (Olson et al., 2021). Therefore, the lateral inhibition standard deviation σ_*x*,*y*_ of each unit that follows the Gaussian distribution (μ_input_ = 0.67,σ_input_ = 0.40, and μ_output_ = 26,*and*σ_output_ = 22) was randomly generated. The setting of lateral inhibition standard deviation in the input layer was inspired by structural characteristics of the visual pathway from the retina to the occipital lobe. The setting of lateral inhibition standard deviation in the output layer was inspired by structural characteristics of the visual pathway from the occipital lobe to the temporal lobe. Although each layer of the model only performs lateral inhibition once, it reflects the result of multiple lateral inhibitions at different levels of the visual pathway. We preliminarily noted that the range of lateral inhibition of various layers of the model was different. The comparison of the simulation results and experimental data revealed that the fitting degree between the simulation results and experimental data is high when the lateral inhibition range of the input layer is large and that of the output layer is small.

The input of each unit of the output layer was equal to the output response of the input layer after

lateral inhibition was multiplied by the weight and summed.


(2) $$b_{i,j}=\sum_{x=1}^{\text{rows}}\sum_{y=1}^{\text{cols}}a_{x,y}^{i\,n\,h}\times w_{x,y,i,j},$$

where *b*_*i,j*_ is the output of the unit in row *i* and column *j* of the output layer without lateral inhibition, $a_{x,y}^{i\,n\,h}$ is the output response of the unit located in row *x* and column *y* of the input layer after lateral inhibition, and *w*_*x,y,i,j*_ is the weight between the unit in row *x* and column *y* of the input layer and the unit in row *i* and column *j* of the output layer.

### Detection of Number Sense

Four control sets were inputted into the neural network, and responses of each unit in the output layer were recorded under 30 repeated runs. If the average response curve of a unit contains the maximum response to a certain numerosity, then, the unit prefers the numerosity. Meanwhile, the distribution of preferred numerosities of numerosity-selective network units at each repeated run was recorded, and the average distribution of preferred numerosities was calculated in the process of 30 repeated runs. First, output responses of units with the same preference (*n* = 1, 2, 4, 6, 8,,28, 30) were averaged to observe whether response curves on the linear scale demonstrate scale and distance effects and obtain average tuning curves. Average tuning curves for network units preferring each numerosity were plotted on a linear scale (*f*(*x*) = *x*). Second, a bar graph was used to plot the average distribution of preferred numerosities obtained under 30 repeated runs to examine the distribution of preferred numerosities of numerosity-selective network units. Finally, the study of the brain showed that response curves of quantity-selective neurons present a positive bias characteristic (Kutter et al., 2018). We fitted Gaussian functions to network tuning curves plotted on a linear scale and three different non-linearly compressed scales $\left(f\,\left(x\right)=x,f\,\left(x\right)=x^{\frac{1}{2}},f\,\left(x\right)=x^{\frac{1}{3}},\mathrm{and}\,f\,\left(x\right)={\text{log}}_{2}\,\left(x\right)\right)$ and calculated the ratio of the regression sum of squares to the total sum of squares to investigate whether tuning curves of the model showed a positive bias characteristic. If the logarithmic scale is suited to tuning curves, then these curves should become symmetric around preferred numerosities when plotted on that scale, and the goodness of fit (*r*-square) of the Gaussian function to tuning curves should be increased. The average goodness of fit to tuning curves on different scales was plotted on a bar graph. We also plotted the standard deviation of the Gaussian function with optimal fit for each tuning curve of numerosity-selective network units on different scales.

We further canceled the lateral inhibition of the input or output layer and observed the output response of the model under the four control sets to analyze the influence of lateral inhibition on the number sense of the two-layered model. First, the lateral inhibition of the input layer was canceled and response curves, distribution of preferred numerosities, and Gaussian fitting results of the model were obtained. The standard deviation σ of the lateral inhibition of the output layer was also set to 0.10, 0.15, 0.20, 0.26, 0.30, 0.35, or 0.40, and the average distribution of preferred numerosities under 30 repeated runs was calculated to explore the influence of the standard deviation on the distribution of preferred numerosities. Second, the lateral inhibition of the output layer was canceled, and the response curve, preference distribution, and Gaussian fitting results of the model were obtained. Based on this, the distribution of preferred numerosities of numerosity-selective network units was obtained when the lateral inhibition standard deviation of the input layer was 0.55, 0.60, 0.65, 0.67, 0.70, 0.75, or 0.80. Finally, the lateral inhibition of input and output layers was canceled, and the response curve, preference distribution, and Gaussian fitting results of the model were obtained.

## Results

### Number Sense Model With Lateral Inhibition in Input and Output Layers

**FIGURE 2:**
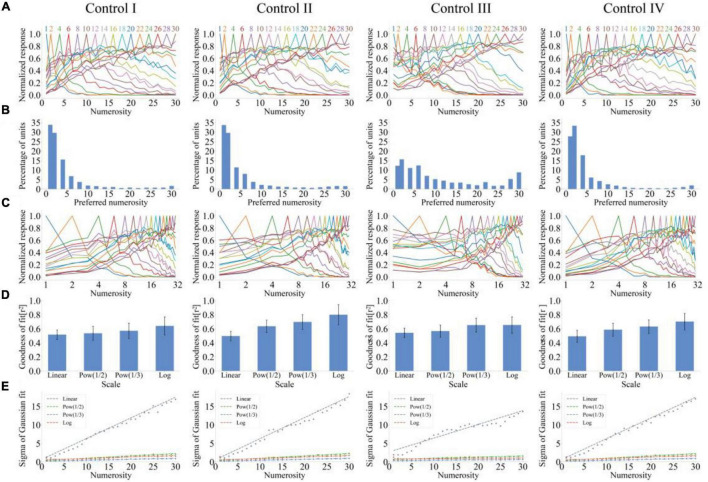
Output response of the lateral inhibition model under four different stimulus sets. **(A)** Average tuning curves for network units that prefer each numerosity plotted on a linear scale. The horizontal axis is the numerosity in the image, and the vertical axis is the average response after normalization. **(B)** Distribution of preferred numerosities of numerosity-selective network units. The horizontal axis is the numerosity, and the vertical axis is the proportion of the number of units that prefer a specific numerosity in the total number of units. **(C)** Average tuning curves for network units that prefer each numerosity plotted on a logarithmic scale. The horizontal axis is the numerosity in the image and plotted on a logarithmic scale of *f*(*x*) = log_2_(*x*), and the vertical axis is the average response after normalization. **(D)** Average goodness-of-fit measure for fitting Gaussian functions to tuning curves on different scales. The average response curves with the preferred numerosity ranging from 1 to 30 were combined *via* Gaussian fitting, and the goodness of fit was calculated using the four scales (*f*(*x*) = *x*^0.5^,*f*(*x*) = *x*^0.33^,*f*(*x*) = *log*_2_(*x*)). **(E)** Standard deviation of the Gaussian function with an optimal fit for each tuning curve of numerosity-selective network units on different scales. The horizontal axis is the preferred numerosity, and the vertical axis is the standard deviation. Left to right images show the results of the output response of the number sense model when four control sets are inputted.

The number sense results when input and output layers of the model demonstrate lateral inhibition are shown in Figure 2. Images from left to right represent numerical abilities of the model when control sets 1–4 are the input. As shown in Figure 2A, the unit produced the maximum response when the input numerosity was equal to the preference numerosity; otherwise, the unit response decreased. A large distance between input and preferred numerosities indicates a small response of the unit. Therefore, the response curve of output units can reproduce the distance effect. Moreover, a large preferred numerosity of the output unit corresponds to a slow decline of the output response when the input numerosity deviates from the preferred numerosity. Therefore, the response curve can reproduce the size effect. The tuning curve of the model conformed to characteristics of “labeled-line code” because it reached the peak at the preferred numerosity instead of monotonically increasing or decreasing.

Figure 2B shows that the number of units with preference numerosity 1 is the largest among the four stimulus sets. The number of units preferring this numerosity decreased as the numerosity increased but increased until the numerosity was close to 30. Therefore, the distribution of preferred numerosities of numerosity-selective network units showed high distribution characteristics at both ends and low distribution characteristics in the middle.

The similarity between response curves and the Gaussian function on different scales requires further investigation for the quantitative exploration of the distance and size effects of output units. Average response curves of the preferred numerosity *n* = 1, 2, 4, 6, 8,28, 30 were plotted on the logarithmic scale (*f*(*x*) = log_2_(*x*), (Figure 2C). The comparison between Figures 2A,C showed that response curves are symmetrical on the logarithmic scale. Figures 2D,E quantitatively demonstrated these observations. Figure 2D presents that the average goodness of fit of response curves on the linear scale was the minimum ($r_{l\,i\,n\,e\,a\,r}^{2}=50.13\%$). The average goodness of fit of the Gaussian fitting of tuning curves increased with the increase of abscissa non-linearity. Figure 2E illustrates that the standard deviation increases with the increase of the preferred numerosity, and the average slope of the four stimulus sets is *r*_*linear*_ = 0.49 on the linear scale. The standard deviation of other non-linear scales was nearly unchanged, and the average slope was *r*_*nonlinear*_ = 0.04. The consistency between these results and experimental data of the prefrontal cortex of the monkey (Nieder and Merten, 2007) indicated that the untrained (i.e., randomly set and unadjusted network connection weights) lateral inhibition model demonstrates number sense.

The lateral inhibition model was compared with other number sense frameworks. A previous study used hierarchical convolutional neural network (HCNN) and showed that numerosity-selective network units spontaneously emerge in a biologically inspired deep neural network (DNN) that was merely trained on visual object recognition (Nasr et al., 2019). However, the HCNN consisted of fifteen layers, namely, one output, eight convolutional, and six pooling layers. Therefore, analyzing the influence of various factors on the number sense is difficult. In addition, although Nasr et al. (2019) proved that numerosity selectivity can emerge simply as a by-product of exposure to natural visual stimuli, without requiring any explicit training for numerosity estimation, they did not study whether the model has number sense before training on visual object recognition. In contrast, DNN of Kim et al. (2021) can spontaneously generate number sense without learning. Numerical abilities of the DNN model can be improved after pretraining. However, this neural network consisted of five convolutional and three fully connected layers; hence, analyzing the visual information process in each layer and distinguishing effects of various factors on numerical abilities are difficult. Zorzi and Testolin (2017) realized the number sense using the genetic algorithm in a three-layered neural network. This finding showed that numerical abilities can be supported by domain-specific representations emerging from evolutionary pressure. However, the input of this model is a binary vector, and determining whether the evolutionary simulation with a realistic sensory input still shows the appearance of numerical cognition is a problem that needs further investigation. Moreover, the model ignores ontogeny of numerical abilities.

In comparison, the proposed lateral inhibition model in this study consists of only two layers of neural networks. Notably, the model can reproduce the distance and size effects when network connection weights are set randomly without adjustment. Compared with other networks (Zorzi and Testolin, 2017; Nasr et al., 2019; Testolin et al., 2020a; Kim et al., 2021), our lateral inhibition model can help analyze the generation of number sense and discuss important factors affecting the number sense.

### Number Sense Model Without Lateral Inhibition in the Input Layer

**FIGURE 3:**
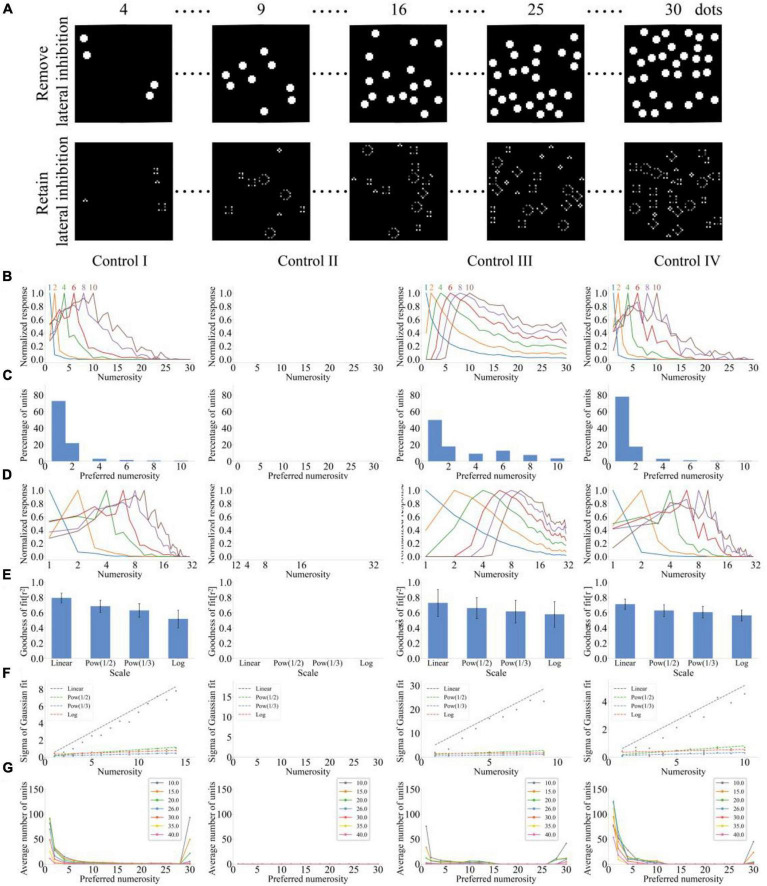
Output response of the neural network model without lateral inhibition in the input layer under four types of stimulus sets. **(A)** Response of the input layer unit in both cases of deleting and retaining the input layer lateral inhibition. The upper layer is the response of the input layer unit after deleting the input layer lateral inhibition. The lower layer is the response of the input layer unit when the input layer presents lateral suppression. **(B)** Average tuning curves for network units that prefer each numerosity plotted on a linear scale. **(C)** Distribution of preferred numerosities of numerosity-selective network units. **(D)** Average tuning curves for network units that prefer each numerosity plotted on a logarithmic scale. **(E)** Average goodness-of-fit measure for fitting Gaussian functions to tuning curves on different scales. **(F)** Standard deviation of the Gaussian function with optimal fitting for each tuning curve of numerosity-selective network units on different scales. **(G)** Distribution of preferred numerosities of numerosity-selective network units under different standard deviations.

The model was tested without input layer lateral inhibition using the stimulus scheme in Figure 2 to analyze the effect of input layer lateral inhibition on number sense. Figure 3A shows the responses of input layer units when the input layer lateral inhibition is canceled or retained. The response of each unit of the input layer was equal to the stimulation intensity of the corresponding pixel in the image when the input layer lacked lateral inhibition. Non-numerical visual features of the item, such as area, shape, perimeter, and radius, were inhibited, and the total response intensity of the input layer was related to the numerosity but unrelated to non-numerical visual characteristics when the input layer demonstrated lateral inhibition. Therefore, the function of input layer lateral inhibition is to extract numerical features of items and suppress non-numerical visual features.

Response curves of units in the output layer without lateral inhibition in the input layer are shown in Figure 3B. The model can sense numerosities less than 10, and average tuning curves fluctuate significantly under the stimulation of control sets 1, 3, and 4. However, the model failed to produce number sense when the total area of items was fixed (i.e., control set 2). Figure 3C presents that more than 50% of units prefer numerosity 1 for control sets 1, 3, and 4. Figure 3D illustrates the response curves of output layer units on the logarithmic scale (*f*(*x*) = log_2_(*x*)). Response curves were more asymmetric on the logarithmic scale compared with those in Figure 3B. We then examined the similarity between response curves and the Gaussian function on different scales. First, the average goodness of fit of the Gaussian function fitting on different scales was calculated (Figure 3E). The results indicated that the goodness of fit on the linear scale increases with the input of control sets 1, 3, and 4 but decreases with the increase of abscissa non-linearity. Second, the standard deviation of the Gaussian function with optimal fitting for each tuning curve of numerosity-selective network units on different scales was calculated (Figure 3F). The results showed that the standard deviation increases with the increase of preferred numerosity on the linear scale but remains unchanged on other non-linear scales. Figure 3G shows the distribution of the number of numerosity-selective units when standard deviations σ of the Gaussian function of the output layer is different. Although the standard deviation σ of the Gaussian function of the output layer changed, the model failed to perceive all numerosities within 30.

The comparison of Figures 2, 3 showed that canceling the lateral inhibition of the input layer changes the response of the model. First, the absence of lateral inhibition in the input layer led to sharp fluctuations in the response curve because failure of this model to inhibit non-numerical visual features of the item results in the reduction of correlation between the total response intensity of the input layer and the numerosity of items. Second, the absence of lateral inhibition in the input layer changed the range of number sense of the model. The preferred numerosity was within 10 for control sets 1 and 4 because the increased intensity of the input stimulation of the output layer subsequently increased the lateral inhibition intensity of output layer units when lateral inhibition was absent in the input layer. Therefore, the response of all units approached zero, and numerosities greater than 10 are not preferred when the number of items was large due to mutual inhibition. The model can also distinguish numerosities within 10 for control set 3 because the total area of the item and the intensity of the input stimulus of the output layer decreased with the increase of the item numerosity when the total circumference of items remained unchanged. Therefore, producing the maximum response to the numerosity exceeding 10 was impossible. The numerical cognitive ability of the model was absent when control set 2 was the input because the intensity of the input stimulation of the output layer and the mutual inhibition intensity of each unit of the output layer remained unchanged when the total area of the item remained the same. Therefore, the response of the output layer unit remained unchanged and failed to produce the maximum response to any number. In summary, number sense is absent in the model without lateral inhibition in the input layer because the model fails to produce number sense stably when controlling the input of non-numerical visual stimulus cues.

### Number Sense Model Without Lateral Inhibition in the Output Layer

**FIGURE 4:**
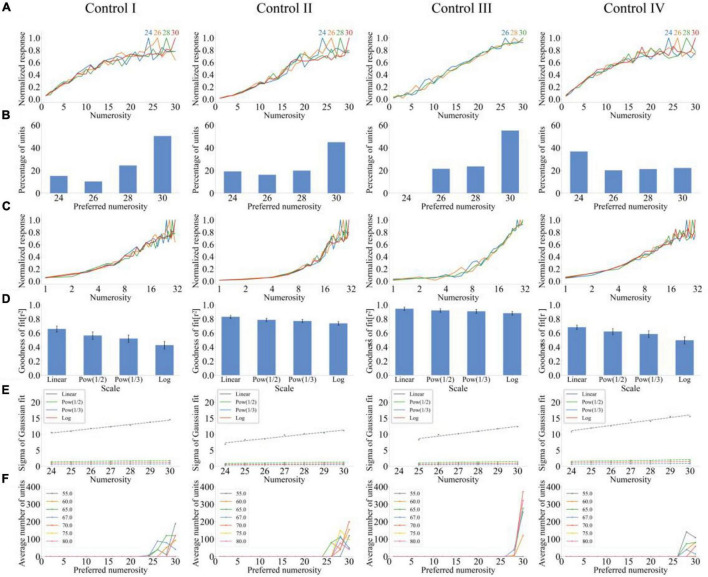
Output response of the model without lateral inhibition in the output layer under four types of stimulus sets. **(A)** Average tuning curves for network units that prefer each numerosity plotted on a linear scale. **(B)** Distribution of preferred numerosities of numerosity-selective network units. **(C)** Average tuning curves for network units that prefer each numerosity plotted on a logarithmic scale. **(D)** Average goodness-of-fit measure for fitting Gaussian functions to tuning curves on different scales. **(E)** Standard deviation of the Gaussian function with optimal fitting for each tuning curve of numerosity-selective network units on different scales. **(F)** Distribution of preferred numerosities of numerosity-selective network units under different standard deviations.

Four types of stimulus sets were used to test the model without output layer lateral inhibition and examine the effect of output layer lateral inhibition on numerical abilities of the model. Average response curves on the linear scale are shown in Figure 4A. The output response of the model increased with the increase of numerosity because the total response strength of the input layer and the response of units in the output layer increased with the increase of numerosity. Figure 4B presents that some units also demonstrate the maximum response to numerosities close to 30. Combined with the influence of the initial weight conforming to the Gaussian distribution, units also preferred numerosities of less than 30. Average response curves on the logarithmic scale (*f*(*x*) = log_2_(*x*)) are shown in Figure 4C. Response curves were fitted with the Gaussian function on linear and non-linear scales. The average goodness of fit of response curves decreased with the increase of the abscissa non-linear scale (Figure 4D). The standard deviation of the Gaussian function with optimal fitting for each tuning curve on different scales (Figure 4E) is qualitatively the same as that in Figure 2E. Figure 4F presents the distribution of preferred numerosities of numerosity-selective network units in the total number of units after adjusting the standard deviation σ of the Gaussian function of the input layer. We revealed that generating the number sense from 1 to 30 was impossible although the standard deviation σ of the Gaussian function of the input layer changed. Notably, failure of the model without output layer lateral inhibition to develop Gaussian-centered tuning curves does not imply that an approximate representation of numerosity is absent in the model. Monotonic tuning curves may be sufficient to encode numerical information coarsely (Chen and Verguts, 2013).

### Number Sense Model Without Lateral Inhibition in Input and Output Layers

**FIGURE 5:**
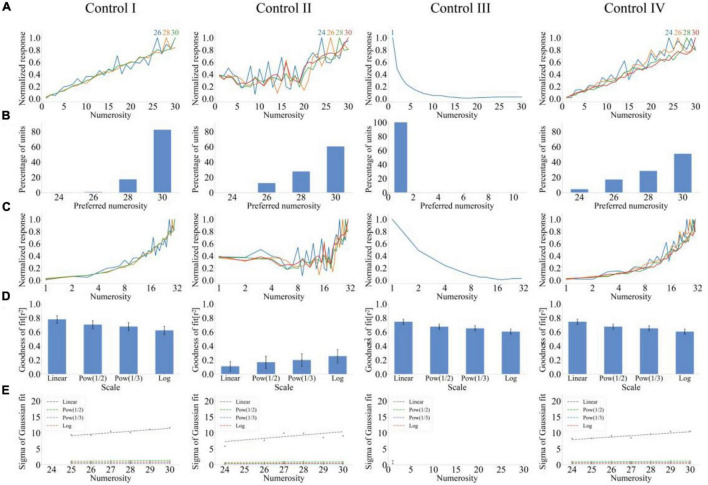
Output response of the neural network model without lateral inhibition in input and output layers under four stimulus sets. **(A)** Average tuning curves for network units that prefer each numerosity plotted on a linear scale. **(B)** Distribution of preferred numerosities of numerosity-selective network units. **(C)** Average tuning curves for network units that prefer each numerosity plotted on a logarithmic scale. **(D)** Average goodness-of-fit measure for fitting Gaussian functions to tuning curves on different scales. **(E)** Standard deviation of the Gaussian function with optimal fitting for each tuning curve of numerosity-selective network units on different scales.

The lateral inhibition function of input and output layers was removed to investigate whether the model without lateral inhibition demonstrates number sense. Average response curves of the model are shown in Figure 5A. Average output responses of the model increased with serious fluctuations with the increase of numerosity. Network units of the output layer demonstrated the maximum response only when the preferred numerosity was close to 30, and control sets 1, 2, and 4 were the input. However, units only preferred numerosity 1 when control set 3 was the input (Figure 5B). Figure 5C presents the average tuning curves for network units that prefer each numerosity. Gaussian fitting was performed on response curves on the four scales. The average goodness of fit increased with the increase of abscissa non-linearity only when control set 2 was the input (Figure 5D). The standard deviation of the Gaussian function with optimal fitting increased with the increase of the preferred numerosity only on the linear scale (Figure 5E). Number sense is absent in the model without lateral inhibition in input and output layers because of the lacking of stable numerical abilities of the model under different control sets.

## Discussion

Compared with other number sense models, the lateral inhibition model presents three main advantages. First, we simplified the number of network layers and parameters. Number sense models typically use complex deep and convolutional neural networks to process complex visual images (Nasr et al., 2019; Testolin et al., 2020b). In contrast, our model used the two-layered neural network to analyze the generation process of number sense. Second, the numerical cognition generation process demonstrated spontaneity. Unlike previous number sense models that rely on quantity-dependent training (Chen and Verguts, 2012, 2017; Miller, 2013; Sengupta et al., 2014), training and transfer learning are unnecessary in our model. The model can reproduce the distance and size effects when network connection weights were set randomly without adjustment. Finally, our model simulated the visual information pathway of newborn animals in a minimalist way. The input layer represents the visual pathway from the retina to the occipital lobe, whereas the output layer represents the visual pathway from the occipital lobe to the temporal lobe. Based on this, we qualitatively added lateral inhibition, which is one of the fundamental mechanisms of the brain. Although models generally adopt the lateral inhibition function (Miller, 2013; Zorzi and Testolin, 2017; Nasr et al., 2019; Kim et al., 2021), they failed to explain the specific role of lateral inhibition in the generation of number sense. We revealed the different effects of lateral inhibition in each layer of the network by selectively removing the lateral inhibition of input and output layers. Lateral inhibition in the input layer inhibits the area, shape, perimeter, radius, and other non-numerical visual characteristics of the item to ensure that the total input layer stimulation intensity is positively correlated with numerosity. Meanwhile, lateral inhibition in the output layer allows output layer units to demonstrate a preference for different numerosities because output responses of units in the output layer are related not only to excitatory stimuli in the input layer but also to lateral inhibition in the output layer. Units located in different locations of the neural network suffer from different degrees of lateral inhibition, given that lateral inhibition intensity is correlated with the Euclidean distance between units. Lateral inhibition is weak when the unit is close to the edge of the neural network. Hence, the numerosity of unit preferences increases as the Euclidean distance between the unit and the geometric center of the neural network increases. In summary, lateral inhibition is an important factor that affects numerical abilities of our two-layered model.

Avoiding the correlation between numerosities and all visual features at the same time and presenting images with the same non-numerical visual features and different numerosities are impossible in the experimental design (Testolin et al., 2020a). Therefore, we adopted the method of maintaining different non-numerical visual features while the numerosity changed to assess whether the number sense of the model was the result of the change of other visual features. Although the lateral inhibition model can generate number sense under these four control sets, numerical processing can be carried out through non-numerical visual features. For example, the model without lateral inhibition in the input layer (Figure 3) can distinguish numerosities within 10 when the total stimulation area was positively correlated with the numerosity. This finding suggested that non-numerical visual features can also influence the number sense of animals. Effects of non-numerical visual features on the response of numerical preference neurons need to be systematically explored in both neurophysiology and deep learning simulation (Leibovich et al., 2017).

Our stimulus sets consisted of customized multiple gray images that aimed to simplify the number of network layers and parameters. Although non-numerical visual stimulus cues were controlled, a gap between stimulus sets and actual visual images still existed. For example, in the actual visual scene, two or more items can overlap. In this case, whether our model can accurately perceive the number of items is unknown. Moreover, items in actual visual images present complex shapes and rich colors. Therefore, testing the number sense of the neural network model on the basis of lateral inhibition in the actual visual scene is necessary. At the same time, our results showed that the simple neural network with lateral inhibition can realize spontaneous numerical cognition without training. However, whether or not number sense can be improved considerably if the neural network is trained is unknown. Moreover, recognition of abstract numbers in our model needs further exploration, given that stimulus sets only contain non-symbolic numerosities.

## Data Availability Statement

The original contributions presented in the study are included in the article/supplementary material, further inquiries can be directed to the corresponding authors.

## Author Contributions

YZ contributed to the conceptualization, data curation, formal analysis, investigation, methodology, and writing of the original draft of the manuscript. HC contributed to the conceptualization as well as writing, review, and editing of the manuscript. YW contributed to the writing, review, and editing of the manuscript. All authors contributed to the article and approved the final version.

## Conflict of Interest

The authors declare that the research was conducted in the absence of any commercial or financial relationships that could be construed as a potential conflict of interest.

## Publisher’s Note

All claims expressed in this article are solely those of the authors and do not necessarily represent those of their affiliated organizations, or those of the publisher, the editors and the reviewers. Any product that may be evaluated in this article, or claim that may be made by its manufacturer, is not guaranteed or endorsed by the publisher.
